# Correction: WWOX promotes osteosarcoma development via upregulation of Myc

**DOI:** 10.1038/s41419-024-06518-8

**Published:** 2024-02-14

**Authors:** Rania Akkawi, Osama Hidmi, Ameen Haj-Yahia, Jonathon Monin, Judith Diment, Yotam Drier, Gary S. Stein, Rami I. Aqeilan

**Affiliations:** 1 The Concern Foundation Laboratories, The Lautenberg Center for Immunology and Cancer Research, Department of Immunology and Cancer Research, IMRIC, Faculty of Medicine, The Hebrew University of Jerusalem, Jerusalem, Israel; 2 Department of Pathology, Hadassah University Medical Center, Jerusalem, Israel; 3 Department of Biochemistry, Larner College of Medicine, UVM Cancer Center, University of Vermont, Burlington, VT USA; 4 Cyprus Cancer Research Institute (CCRI), Nicosia, Cyprus

**Keywords:** Bone cancer, Cell growth

Correction to: *Cell Death and Disease* 10.1038/s41419-023-06378-8, published online 05 January 2024

In this article the third author name has been given erroneously as Ameen Haji Yehya.

It should be read: Ameen Haj-Yahia.

The original article has been corrected.

