# Supplementary material for: WWOX promotes osteosarcoma development via upregulation of Myc
Source: Cell Death Dis. 2024 Jan 5;15(1):13. doi: 10.1038/s41419-023-06378-8 (PMC10770339; doi:10.1038/s41419-023-06378-8)
Supplement: Supplementary file 2 — Original Western blot Data File [file 41419_2023_6378_MOESM2_ESM.pptx]

## Slide 1
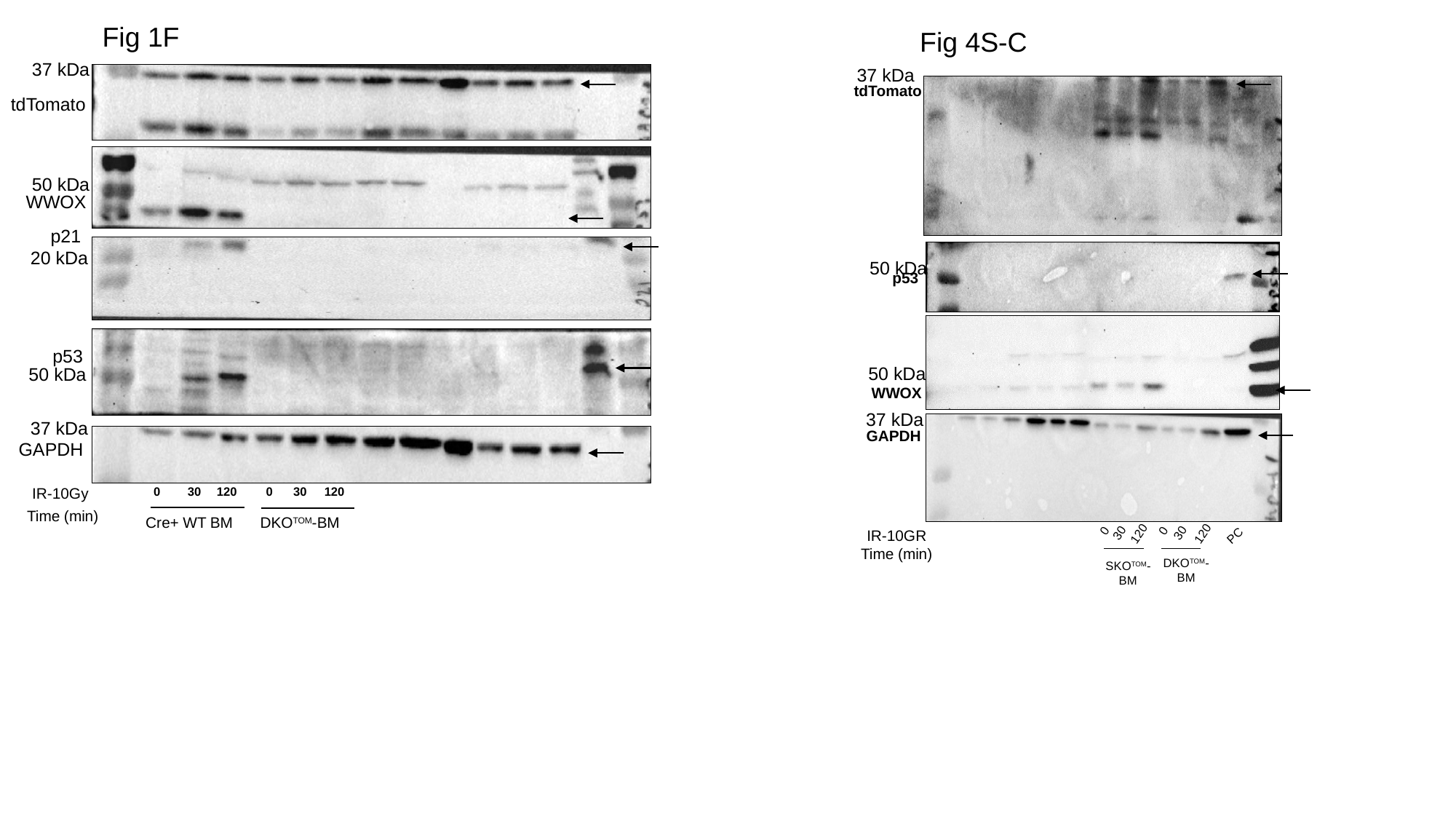

Fig 1F
Fig 4S-C
37 kDa
37 kDa
tdTomato
50 kDa
p53
50 kDa
WWOX
37 kDa
GAPDH
0
0
30
30
120
120
IR-10GR
Time (min)
PC
DKOTOM-BM
SKOTOM-BM
tdTomato
50 kDa
WWOX
p21
20 kDa
p53
50 kDa
37 kDa
GAPDH
IR-10Gy
0
30
120
0
30
120
Time (min)
Cre+ WT BM
DKOTOM-BM

## Slide 2
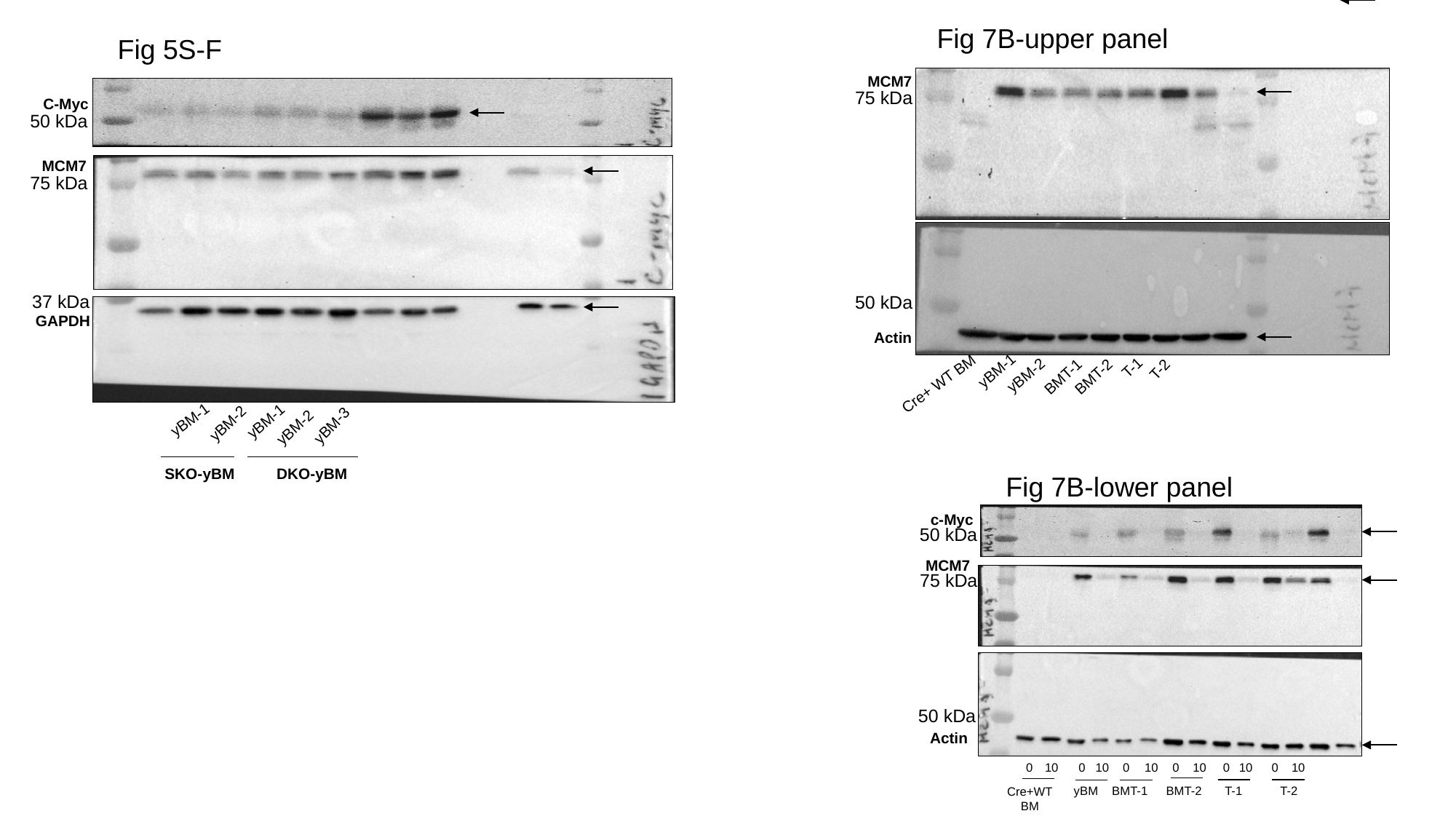

Fig 7B-upper panel
Fig 5S-F
MCM7
75 kDa
50 kDa
Actin
BMT-1
T-2
T-1
yBM-1
yBM-2
BMT-2
Cre+ WT BM
C-Myc
50 kDa
MCM7
75 kDa
37 kDa
yBM-1
yBM-1
yBM-2
yBM-3
yBM-2
DKO-yBM
SKO-yBM
GAPDH
Fig 7B-lower panel
c-Myc
50 kDa
MCM7
75 kDa
50 kDa
Actin
10
10
10
10
10
10
0
0
0
0
0
0
BMT-2
yBM
BMT-1
T-1
T-2
Cre+WT
BM

## Slide 3
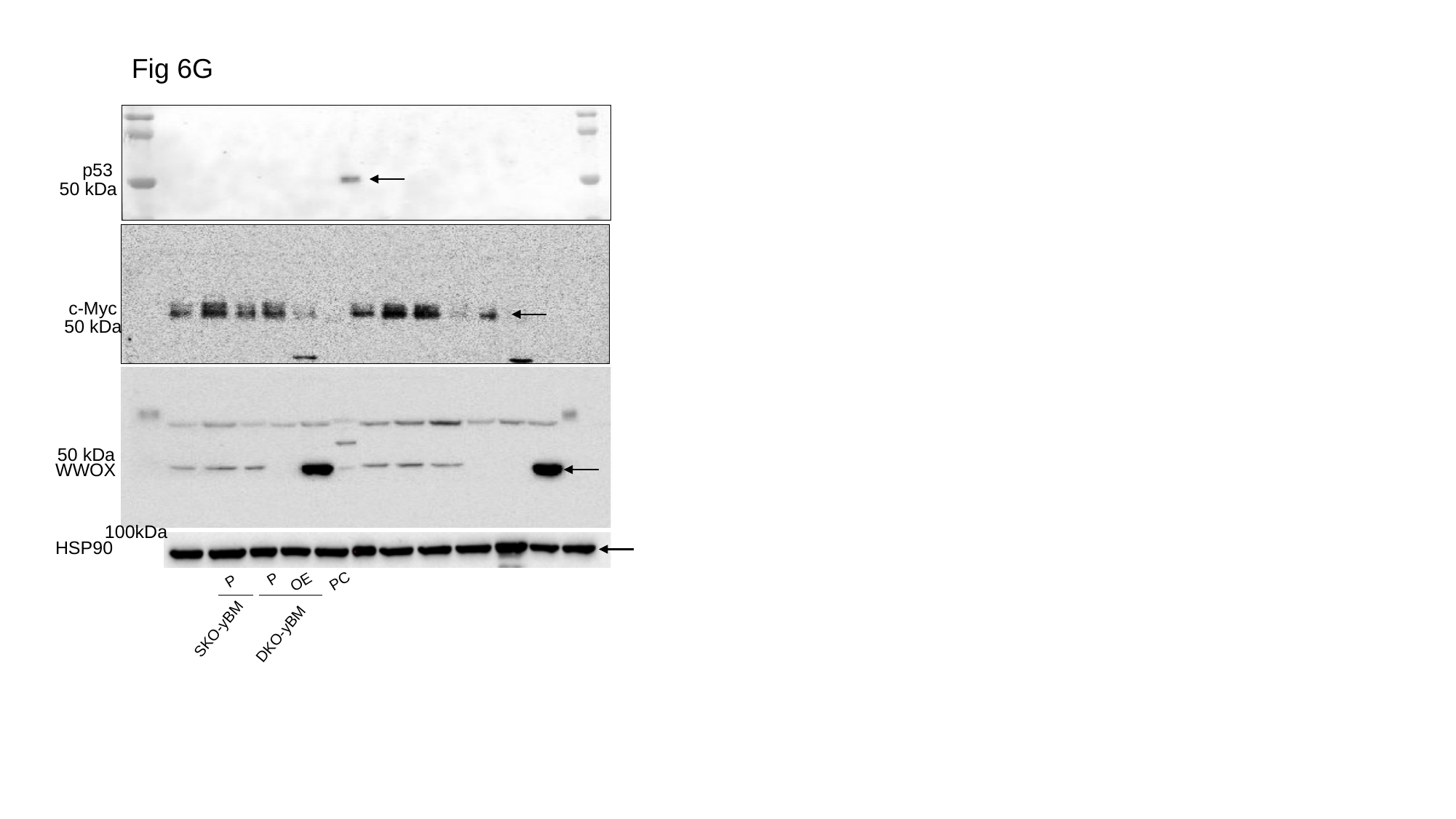

Fig 6G
p53
50 kDa
c-Myc
50 kDa
50 kDa
WWOX
100kDa
HSP90
P
PC
P
OE
SKO-yBM
DKO-yBM
